# Bioinformatics analysis of immune-related prognostic genes and immunotherapy in renal clear cell carcinoma

**DOI:** 10.1371/journal.pone.0272542

**Published:** 2022-11-23

**Authors:** Ziwen Pan, Sheng Chang, Song Chen, Daqiang Zhao, Zhiyu Zou, Linrui Dai, Yibo Hou, Qianqian Zhang, Yuanyuan Yang, Zhishui Chen, Weijie Zhang, Yuanyuan Zhao

**Affiliations:** Institute of Organ Transplantation, Tongji Hospital, Key Laboratory of Organ Transplantation, Ministry of Education, NHC Key Laboratory of Organ Transplantation, Key Laboratory of Organ Transplantation, Chinese Academy of Medical Sciences, Tongji Medical College, Huazhong University of Science and Technology, Wuhan, China; The First Affiliated Hospital of Nanjing Medical University, CHINA

## Abstract

Clear cell renal cell carcinoma (ccRCC) is an immunogenic tumor, and investigating the immunorelated genes is essential. To investigate the immunoprognostic genes of ccRCC, we analyzed the data assimilated from a public database (The Cancer Genome Atlas (TCGA) database and the gene expression omnibus (GEO) database) using bioinformatics. Then, an immunoprognosis model was constructed to identify four hub genes with moderate predictive values for the prognosis of ccRCC patients. These four genes were associated with the prognosis of ccRCC patients based on Oncomine and Gena Expression Profiling Interactive Analysis (GEPIA) databases. The correlation analysis between the immune infiltrate, immune checkpoints, and immunotherapy and this immunoprognosis model showed that immune infiltration could predict the immunotherapy effects. We also conducted a quantitative real-time polymerase chain reaction analysis and found that the expressions of three hub genes were associated with tumor progression (*P*<0.1). In conclusion, four genes that may serve as potential biomarkers in ccRCC were identified with respect to prognosis.

## Introduction

ccRCC is an immunogenic tumor and benefits from immunotherapy. Ipilimumab, an anti-CTLA-4 antibody, combined with nivolumab, showed a response rate of about 40% in the CheckMate 016 trial [1]. Recent advances in bioinformatics have focused on the correlation between ccRCC prognosis and immunity. The targeted immune cell checkpoints are one of the most effective methods to activate an anti-tumor immune response. Cytotoxic T lymphocyte-associated protein 4 (CTLA-4) and programmed cell death protein 1 (PD-1) are two common immune checkpoints. Several studies reported immuoprognostic models (IPMs). Luo et al. [2] revealed three IPMs to assess the prognosis of metastatic colorectal cancer, obtained at 1, 3, and 5 years; the area under the curve (AUC) was 0.646, 0.7, and 0.692, respectively. Moreover, the tumor-infiltrating immune cells (TIICs) [3] are crucial in modulating tumor progression and have a potential prognostic value. A previous study showed that intratumoral immune cell infiltration is associated with the outcomes of cancer patients. Choueiri et al. [4] reported that kidney cancer patients with a high abundance of CD8+T cells have a poor prognosis, while Bromwich et al. [5] indicated that CD4+T cell infiltration was associated with poor survival. Presently, the mechanism of how immunity affects the occurrence and development of ccRCC is unclear. Thus, the current study aimed to establish a correlation between immunity and the occurrence and development of ccRCC. This study analyzed the immune-related differentially expressed genes (DEGs) associated with ccRCC prognosis. These findings would be beneficial in improving the prognosis of patients with ccRCC.

## Materials and methods

### Data and database analysis

Data were obtained from TCGA and GSE53757 in the GEO database [6]. The GSE53757 source files were transformed into log2 and normalized to expression matrices. DEGs were calculated using the R package “Limma” to differentiate between ccRCC and normal kidney samples. Subsequently, the DEGs were screened if the log2fold-change was >1 and the false discovery rate (FDR) was 0.05. TCGA data after pretreatment using the “edgeR” R package for variance analysis were set to several times the expression of genes, the amount of correction after each gene expression to >1, the domain value as |log2fold-change|>2, and FDR<0.05 for screening the DEGs. The “VennDiagram” package was used to identify the intersection of the DEGs obtained from TCGA and GEO databases and the immune-related genes (IRGs) downloaded from the Import database. The study was conformed to the guidelines set forth by the Declaration of Helsinki. Written informed consent was obtained from all participants. The study was approved by the appropriate local institutional review boards on human research at the Tongji Hospital, Tongji Medical College, Huazhong University of Science and Technology (IRB No.: TJ-IRB20220106). Verbal informed consent

### Function enrichment analysis of DEGs

We used the R packages of “clusterprofiler,” “enrichplot,” and “ggplot2” for Gene Ontology (GO) and Kyoto Encyclopedia of Genes and Genomes (KEGG) pathway analysis. Significant *P*- and *Q*-values were set to 0.05.

### Prognostic-related survival model

Using IRGS, univariate Cox risk regression, and least absolute shrinkage and selection operator (LASSO) regression analysis (Simple Random sampling of “caret” package with R software was performed at the ratio of 2:1 for the training and test groups), multivariate Cox risk regression was analyzed to build a risk prognosis model, and survival-related hub genes were screened out. *P*<0.001 was statistically significant in the univariate Cox regression. The “Glmnet” package of R was used to set 1000-fold replacement and 10-fold cross-validation for LASSO regression. Kaplan–Meier survival plots showed the prognosis of ccRCC patients. the AUC was calculated using the R package “survivalROC.”

### Oncomine and GEPIA databases [7] were used to analyze the prognostic value of DEGs

In order to verify the hub genes, five qualified databases were obtained from Oncomine [8] (www.oncomine.org/), followed by a meta-analysis. The filter conditions were as follows: the cancer type was ccRCC, the sample type was mRNA, and the analysis type was a case-control study. The threshold was set at 1e-4, the fold-change to 2, and the gene ranked up to 10%. Then, the GEPIA database (http://gepia.cancer-pku.cn/) was analyzed to determine the survival prognosis of hub genes.

### Immunoinfiltration analysis of prognostic genes

Cibersort [9] is a deconvolution algorithm that quantifies the cell components from a large number of tissue gene expression profiles. Because of prior knowledge of the expression profiles of purified leukocyte subpopulations, Cibersort can accurately estimate the tumor tissue’s immune composition.

### Genetic alteration in genes key related to prognosis

The mutation data were obtained from the TCGA database. Next, we analyzed the genetic changes in different risk groups and their correlation with prognosis.

### Immunotherapy and immune targets of prognostic genes associated with ccRCC

We investigated the differential expression of *PD-1* and *CTLA-4* genes in patients in different risk groups. In addition, we downloaded 4 (kidney renal clear cell carcinoma) KIRC files related to immunotherapy from the cancer immunoAtlas [10] (https://tica.at/). These included ctla4_neg_pd1_neg, ctla4_neg_pd1_pos, ctla4_pos_pd1_neg, and ctla4_pos_pd1_pos. Under the four conditions above, the Wilcoxon test was used to evaluate the efficacy of immunotherapy in the two risk groups.

### RNA extraction and quantitative real-time polymerase chain reaction (qRT-PCR)

In this study, 12 pairs of ccRCC and para-cancerous tissues were collected from Tongji Hospital of Huazhong University of Science and Technology (Wuhan, China) between January 2022 and May 2022. Total RNA was isolated from ccRCC and normal kidney cells using RNA-easy^TM^ Isolation Reagent Vazyme Cat (Vazyme, Nanjing, China). A reverse transcription kit (Vazyme) was used to convert the mRNA into cDNA. qRT-PCR was carried out with a SYBR Green kit R701-02 (TransGen Biotech, Beijing, China) in a final volume of 10 mL to detect the expression level of these genes. The data were analyzed using StepOne software (Thermo Fisher Scientific, China). *GAPDH* was used as a reference gene to calculate the relative expression levels using 2^-DDCT^. Primer sequences are listed in the S1 Table.

### Statistical analysis

R packages “edgeR” and “Limma” were used to analyze the differential genes. Two groups were compared using the Wilcoxon test. Survival analysis was carried out using Kaplan–Meier method and the log-rank test. The survival-related receiver operating characteristic (ROC) package was utilized to draw the ROC curve. The chi-square test was used to analyze the differences between the two groups in terms of clinical parameters, and the difference was considered statistically significant at *P*<0.05. All statistical analyses were performed using GraphPad Prism 9 software (GraphPad Software, San Diego, CA, USA).

## Results

### Characteristics and functional clustering of 128 differentially expressed IRGs in ccRCC

**Fig 1:**
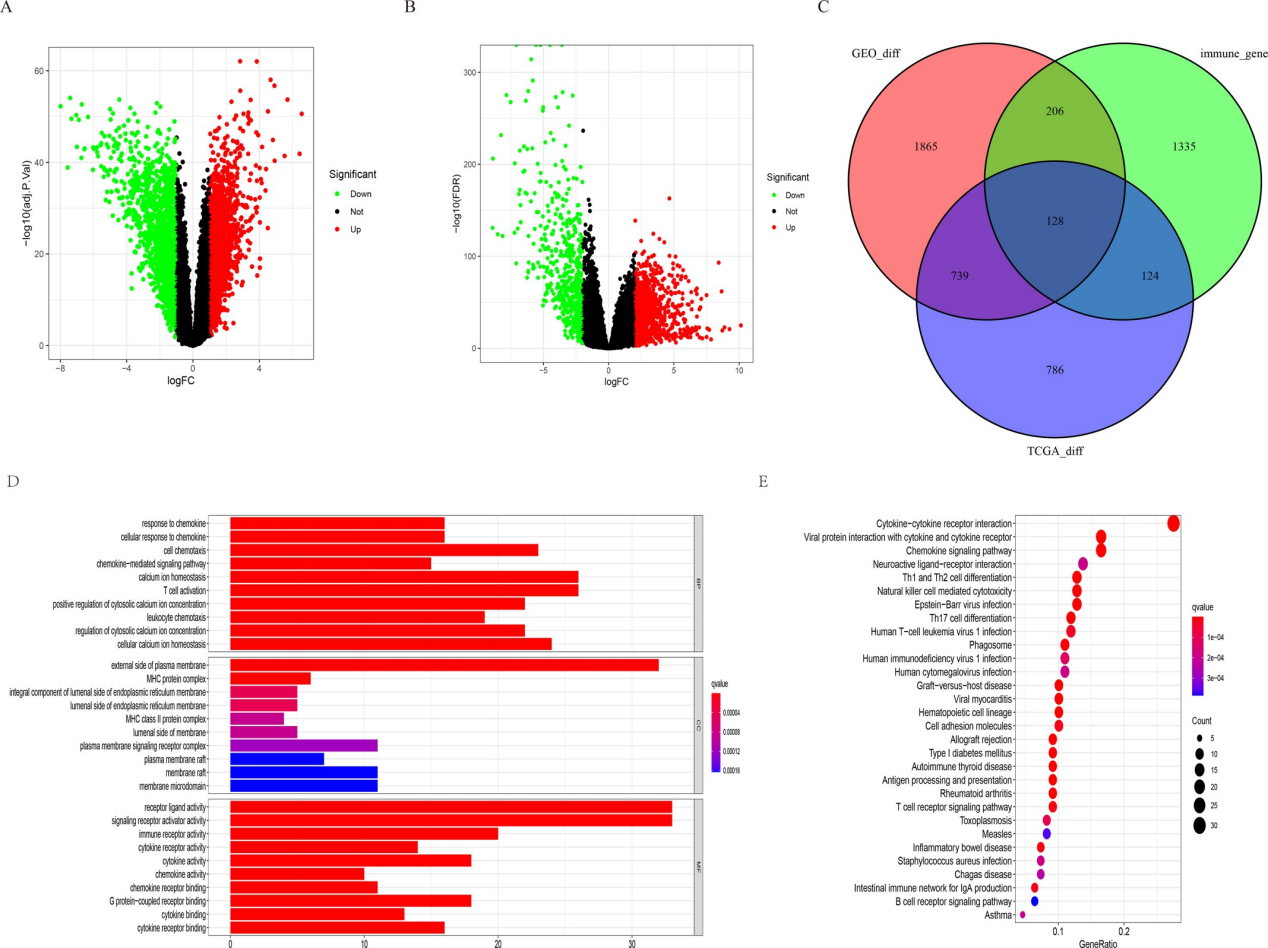
Differentially expressed IRGs. (A-B) represents the volcano map of DEGs from the TCGA and GSE53757 datasets. (C) Venn diagrams of DEGs in TCGA and GSE537357 dataset and IRGs. (D-E) GO and KEGG enrichment analysis of differential expression of IRGs. BP, biological process. CC, cellular component; MF, molecular function.

**Fig 2:**
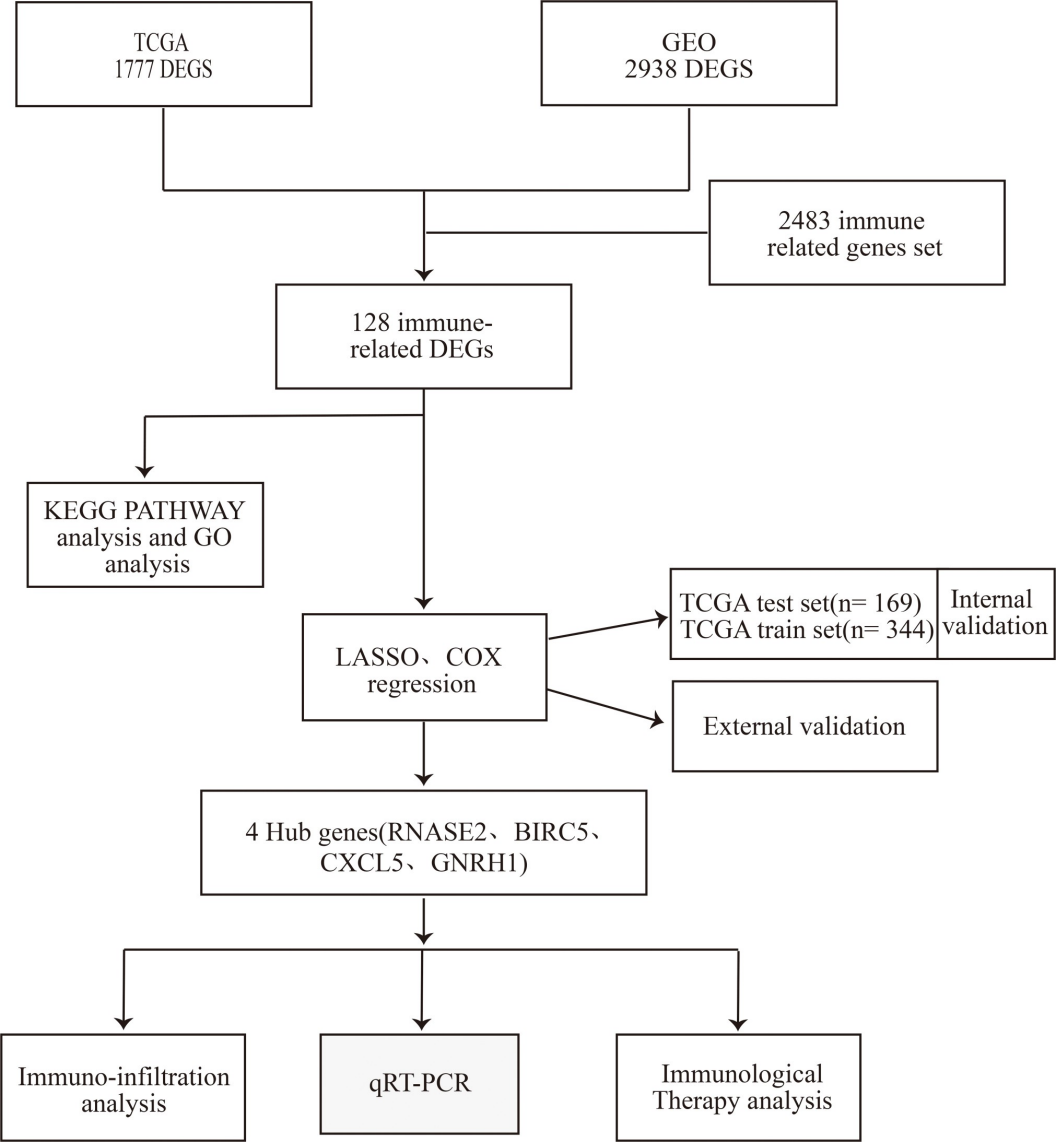
Schematic of the study process.

A total of 1777 DEGs were obtained from the TCGA database (Fig 1A), and 2938 DEGs were obtained from the GEO database (Fig 1B). These were pooled with 2483 IRGs, and finally, 128 IRGs (Fig 1C and Table 1) overlapping in both databases were identified, including 102 upregulated and 26 downregulated IRGs. GO (Fig 1D) and KEGG (Fig 1E) enrichment analyses of the above IRGs showed a response to chemokines, external side plasma membrane, and receptor-ligand activity, which are the most common biological terms for BP, CC, and MF. The most enriched form in KEGG pathway analysis was the cytokine-cytokine receptor interaction. The study schematic is illustrated in Fig 2.

**Table 1:** 128 IRGs.

*ACKR3*	*CARD11*	*CHGB*	*ESRRG*	*HLA-DPB1*	*IL1RL1*	*LTB4R*	*PRF1*
*ADM*	*CCL18*	*CHIT1*	*FABP6*	*HLA-DQA1*	*IL20RB*	*LYZ*	*PTGDR*
*ANGPTL1*	*CCL20*	*CR2*	*FABP7*	*HLA-DQB1*	*IL21R*	*MARCO*	*PTGER1*
*ANGPTL4*	*CCL4*	*CXCL10*	*FAM3B*	*HLA-F*	*IL2RA*	*MCHR1*	*PTGER3*
*APLN*	*CCL5*	*CXCL11*	*FASLG*	*HLA-G*	*IL2RB*	*MMP9*	*PTH1R*
*APOBEC3C*	*CCR5*	*CXCL13*	*FCER1G*	*HMOX1*	*INHBB*	*MSR1*	*PTHLH*
*APOBEC3G*	*CD1D*	*CXCL5*	*FCGR2B*	*HSPA2*	*INHBE*	*NMB*	*RAC2*
*AQP9*	*CD244*	*CXCL9*	*FGF1*	*HSPA6*	*ISG20*	*NOD2*	*REG1A*
*AVPR1B*	*CD247*	*CXCR3*	*FGF9*	*ICOS*	*ITGAL*	*NOS1*	*RNASE2*
*BDNF*	*CD3D*	*CXCR4*	*GBP2*	*IDO1*	*ITGB2*	*NR0B2*	*S100A14*
*BIRC5*	*CD3E*	*CXCR6*	*GCGR*	*IGHM*	*ITK*	*NR1I3*	*S100A2*
*BMP7*	*CD3G*	*DLL4*	*GNLY*	*IGKC*	*KLRD1*	*NR2E1*	*SCG2*
*BMPR1B*	*CD70*	*EBI3*	*GNRH1*	*IGLV1-44*	*KNG1*	*PAEP*	*SEMA5B*
*BTK*	*CD72*	*EGF*	*GZMB*	*IL10RA*	*LAT*	*PDGFRA*	*SEMA6D*
*C3*	*CD86*	*ESM1*	*HCK*	*IL12RB1*	*LCP2*	*PF4V1*	*SH2D1A*
*CALCA*	*CD8A*	*ESRRB*	*HLA-DOB*	*IL12RB2*	*LILRB3*	*PIK3R5*	*STC2*

### Establishment and validation of prognostic risk models of the four hub genes

**Fig 3:**
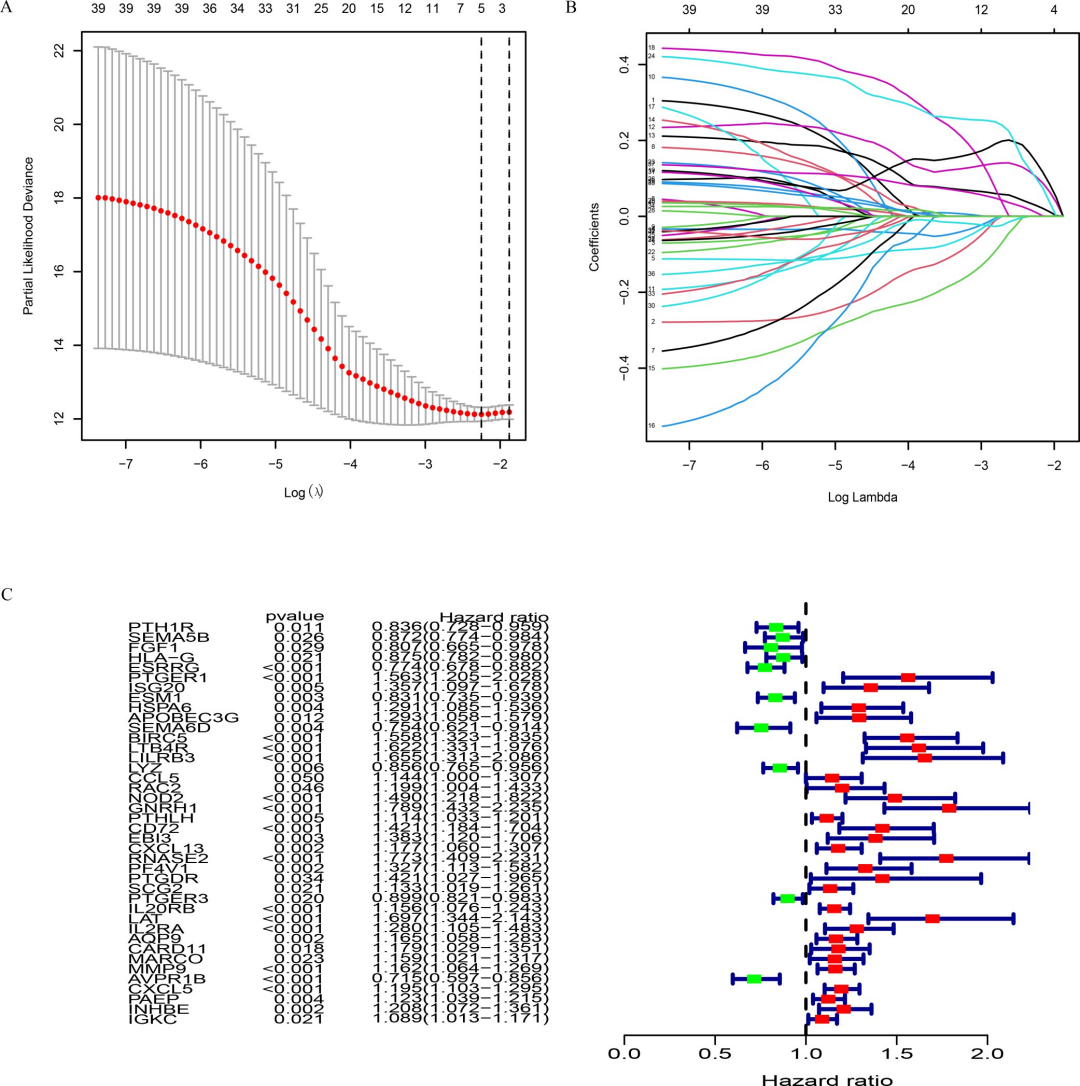
LASSO regression and univariate Cox regression. A Forest plot consists of 21 IRGs. (B-C) LASSO regression model.

In order to identify the key IRGs in the occurrence and development of ccRCC and use them as potential predictive targets, 128 IRGs were evaluated by univariate Cox regression analysis. Among these, 40 DEGs related to survival were screened out, and a forest plot was constructed (*P*<0.001; Fig 3A). LASSO regression analysis (Fig 3B and 3C) was performed, and five DEGs were obtained. Then, multivariate Cox regression analysis was performed, and four prognostic genes were screened out: *BIRC5*, *GNRH1*, *RNASE2*, and *CXCL5* (Table 2). Immune-related gene-based prognostic index (IRGPI) was calculated according to the risk score (Table 2). The following formula was applied to determine risk score of the patients:

Risk score = (0.219681×BIRC5)+(0.492465×GNRH1)+(0.383803×RNASE2)+(0.096701×CXCL5).

**Table 2:** Multivariate Cox regression analysis revealed four prognostic markers genes.

Gene symbol	Coef	HR	HR.95L	HR.95H	*P*-value
*BIRC5*	0.219681	1.24568	1.035347	1.498741	0.019907
*GNRH1*	0.492465	1.636344	1.301839	2.056801	2.44E-05
*RNASE2*	0.383803	1.467856	1.147024	1.878426	0.002288
*CXCL5*	0.096701	1.101531	1.007325	1.204547	0.034009

**Fig 4:**
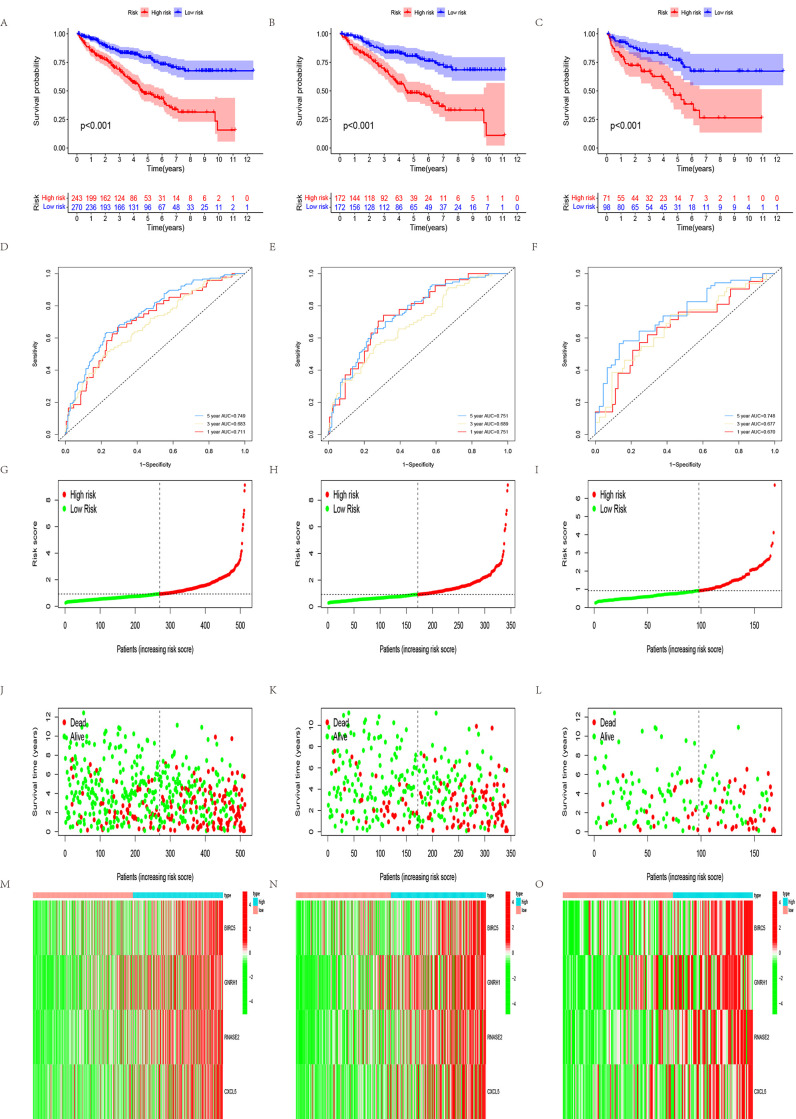
Survival models and internal validation of prognostic IRGPI for all patients with ccRCC, training and test groups, and correlation between risk scores and clinical traits. (A–C) Three groups of patients had good prognoses in the low-risk group, respectively. (D–F) ROC curves of the prognostic survival model of the three groups. (G–I) Risk score of the high- and low-risk groups in the three groups. (J–L) survival time and status of patients in the three groups. (M–O) Heat map of the four hub genes between the high- and low-risk groups.

**Fig 5:**
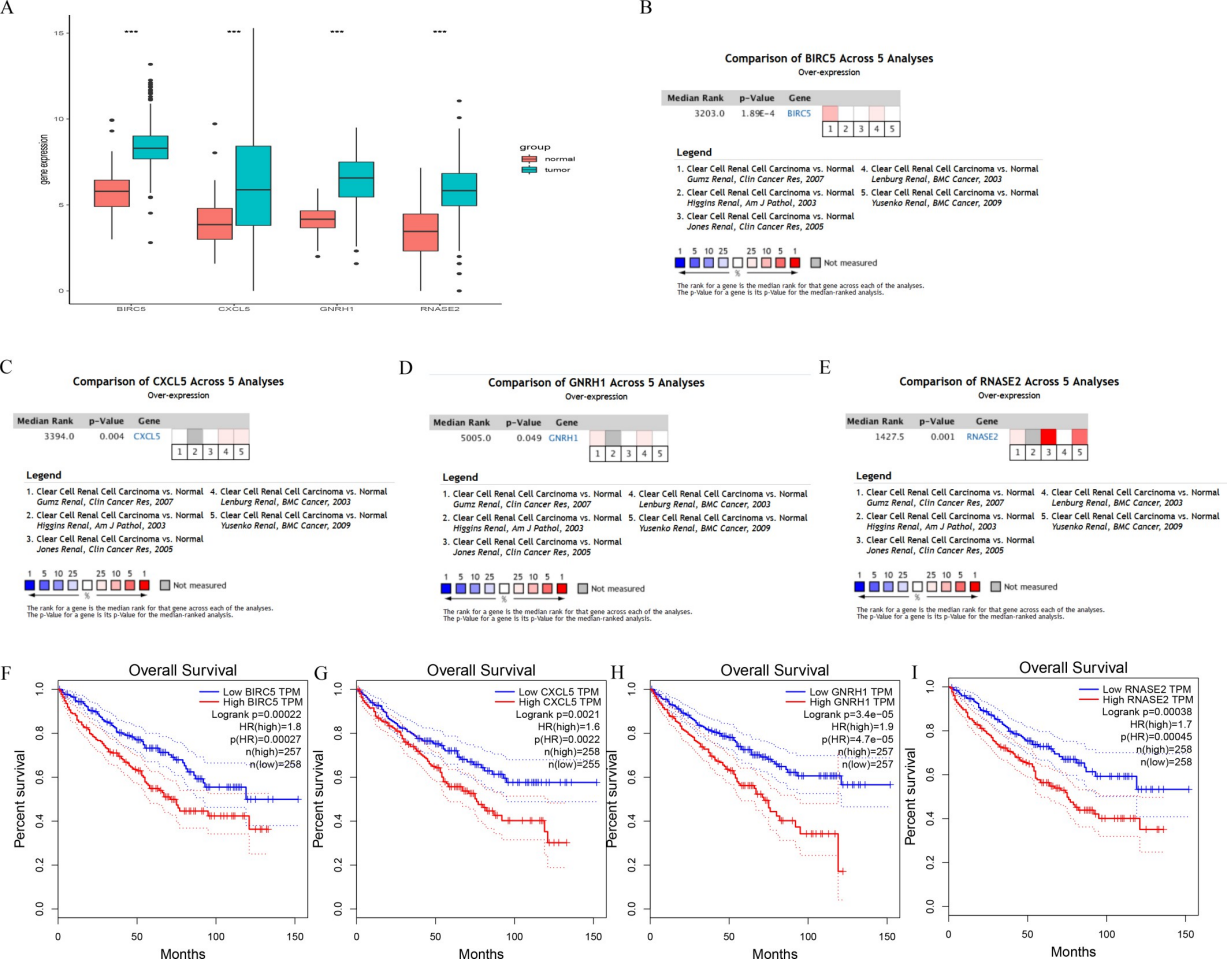
Differential expression and external validation of four hub genes between tumor and normal groups. (A) boxplot of the differential expression of four hub genes in TCGA. (B–E) external validation using the Oncomine database. (F–I) expression of the genes was negatively correlated to tumor prognosis (***P<0.001).

The median of the risk score calculated using the IRGPI-based risk prognosis model was 0.9243977, and the samples downloaded from the TCGA database were divided into low- and high-risk groups. To verify the reliability of the model, we analyzed the outcomes and found that the low-risk group had better outcomes than the high-risk group (*P*<0.001; Fig 4A–4C). The ROC curve showed that our models have a moderate predictive value (Fig 4D–4F). We also analyzed the correlation between four hub genes and clinically relevant indicators and found that the gender, tumor grade, and tumor stage were correlated with risk score (*P*<0.05; S2B–S2D Fig). Additionally, the four hub genes were significantly upregulated in the tumor group based on the GEO database (*P*<0.001; Fig 5A). Then, we conducted a meta-analysis of four hub genes based on the results from the Oncomine database, and the results were consistent with previous results (*P*<0.05; Fig 5B–5E). To verify whether these four hub genes are related to prognosis, survival analysis was performed using GEPIA tools (http://gepia.cancer-pku.cn/); the expression of the genes was negatively correlated to tumor prognosis (*P*<0.05; Fig 5F–5I).

### Characteristics of immune invasion in the two risk groups were significantly different

**Fig 6:**
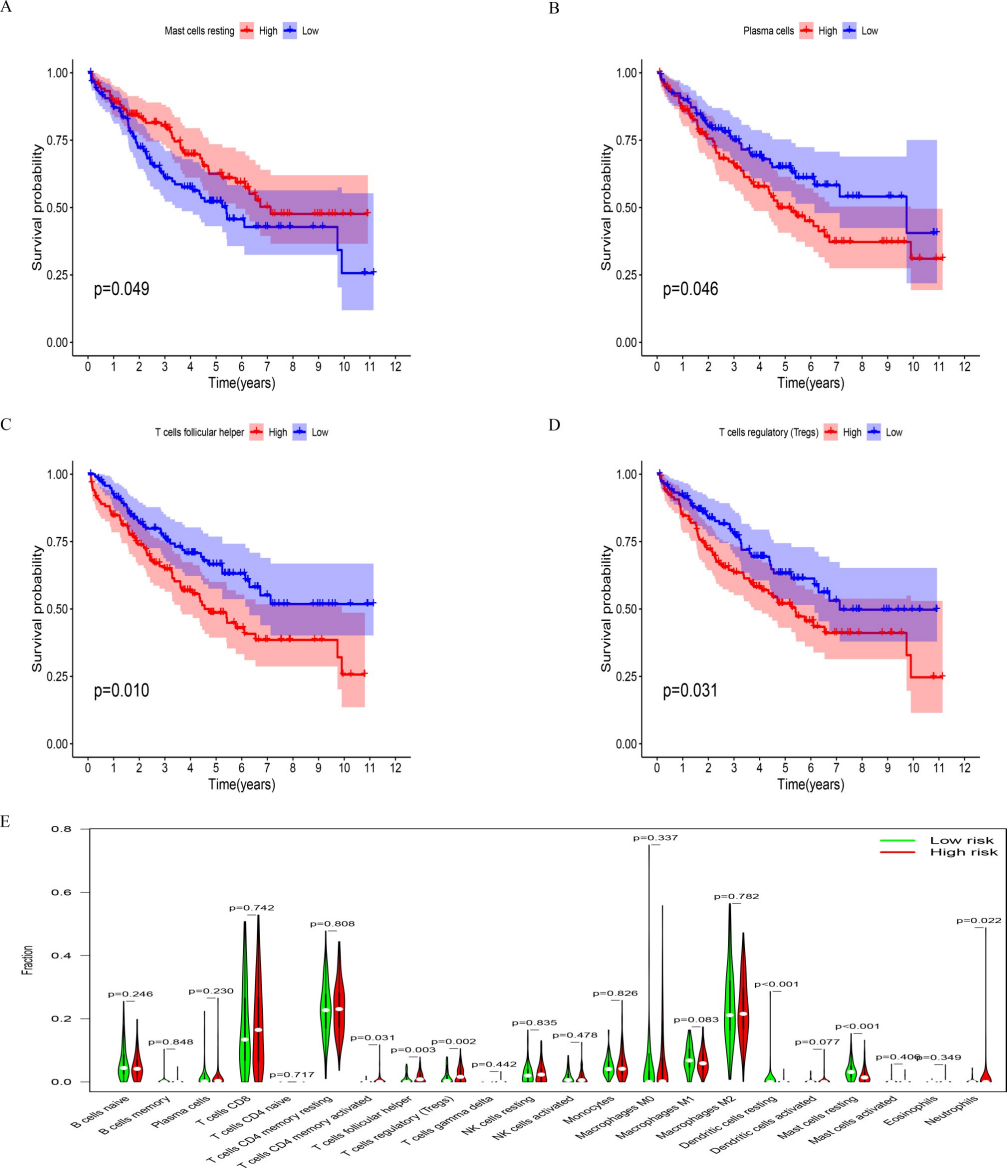
Comparison between two risk groups and the immune cell content and prognosis of immune cells. (A) difference analysis of immune cell content in the two risk groups. (B–E) Prognostic analysis of patients with a varying number of resting mast cells, plasma cells, follicular helper T cells, and regulatory T cells.

The evaluation of the two risk groups of patients and correlation immunity infiltration (Fig 6A) revealed that the high-risk group had higher levels of CD4 memory T cells, follicular helper T cells, and regulatory T cells, Neutrophils, while the low-risk group had higher levels of resting dendritic cells, resting mast cells. Moreover, we analyzed the prognosis of immune cell content in the two risk groups (Fig 6B–6E) and found that patients with high resting mast cells, low plasma cells, low follicular helper T cells, and low regulatory T cells showed improved prognosis. Moreover, These data suggested that the immune status of ccRCC can be predicted based on four hub genes.

### Mutation load was significantly different between the two risk groups

A high mutation load and increase in neoantigens were associated with the clinical effects of the immune checkpoint inhibitor therapy. Therefore, we analyzed the genetic changes in the two risk groups of samples (S2A and S2B Fig) and found the primary mutation in the high-risk group in Missense_Mutation, In_Frame_Del, Frame_Shift_Ins, Translation_Start_Site, Frame_Shift_Del, In_Frame_Ins, Nonsense_Mutation, and Multi_Hit in three genes, *VHL*, *PBRM1*, and *SETD2*. The main mutation points in the low-risk group were Nonsense_Mutation, Translation_Start_Site, Frame_shift_Del, In_Frame_DeI, Frame_Shift_Ins, Nonstop_Mutation, Missense_Mutation, and Multi_Hit in three genes: *VHL*, *PBRM1*, and *TTN*. We also analyzed the differences in the tumor mutation load expression in the two groups (S2C Fig) and found that the high-risk group had a higher tumor mutation load than the low-risk group.

### Expression of immunotarget-related genes and the efficacy of immunotherapy differ significantly in the two risk groups

**Fig 7:**
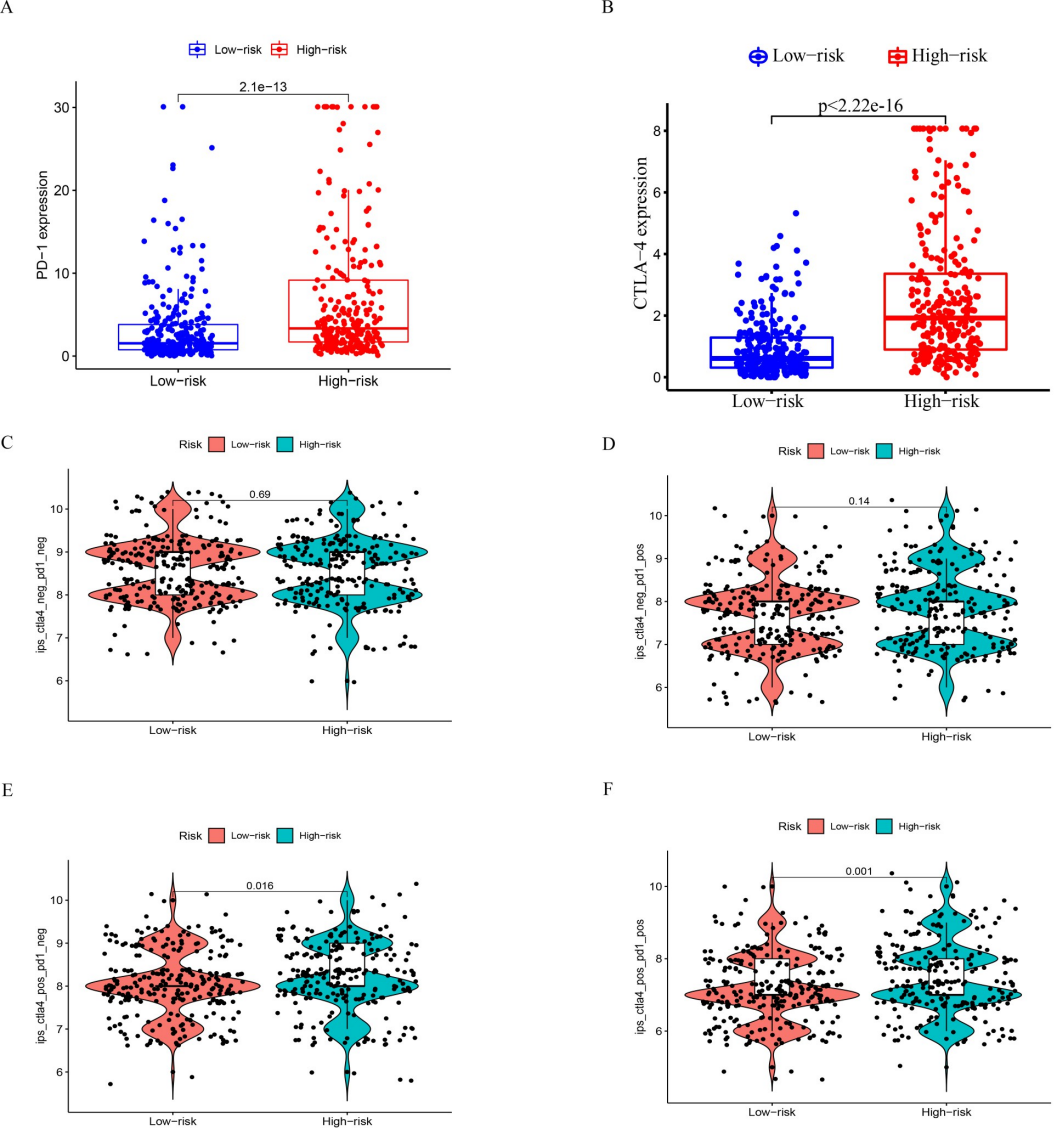
Differential expression of *PD-1* and *CTLA-4* and related immunotherapy in patients in the two risk groups. (A-B) represents the differential expression of *PD-1* and *CTLA-4* in the two risk groups. (C–F) represents the difference analysis of patients in the two risk groups when receiving immunotherapy for anti-PD-1 and CTLA-4.

We analyzed the differential expression of *PD-1* and *CTLA-4* in both groups and found that *PD-1* and CTLA-4 were overexpressed in the high-risk group (Fig 7A and 7B). Next, to demonstrate the effectiveness of our survival prognosis model, we analyzed the efficacy of immunotherapy in the two risk groups under four different conditions (Fig 7C–7F). The immunotherapy efficacy of patients in the high-risk group was significantly stronger when they received either CTLA-4-positive or both treatments. The findings indicated that immunotherapy is more effective in high-risk ccRCC than in the low-risk group.

### Expression of four hub genes by qRT-PCR at the cellular and tissue levels of ccRCC

**Fig 8:**
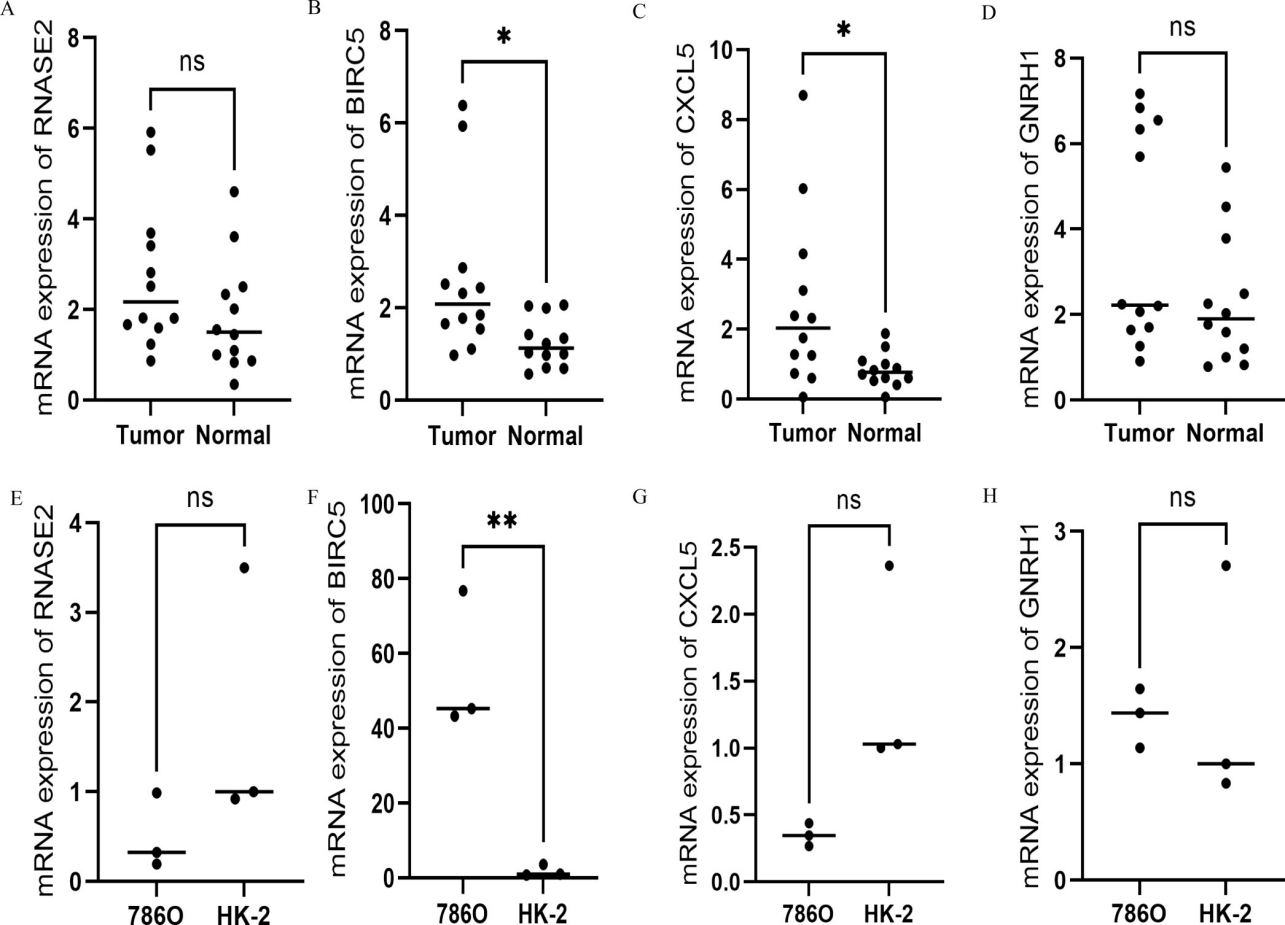
The mRNA levels of the four hub genes. (A–D) Expression of the four genes in 12 pairs of ccRCC and para-cancer tissues by qRT-PCR. (E–H) expression of the four hub genes in cancer cells and normal kidney cells by qRT-PCR (**P<0.01; *P<0.05).

According to qRT-PCR analysis, *BIRC5* and *CXCL5* mRNA levels were significantly higher in ccRCC than in the paracancer tissues (*P*<0.05; Fig 8B–8C). Furthermore, compared to the normal renal epithelial cell line HK-2, the ccRCC cell line 786O expressed significantly more *BIRC5* (*P*<0.01; Fig 8F). Due to the short postoperative follow-up time of the collected cases, we analyzed the correlation between the qRT-PCR expression values of the four hub genes and clinicopathology (Table 3) and found that the expression values of three hub genes (*RNASE2*, *BIRC5*, and *GNRH1*) were associated with tumor progression (*P*<0.1).

**Table 3:** Correlation between the expression levels of four hub genes and clinicopathology.

Characteristic	*RNASE2*		*P*-value	*BIRC5*		*P*-value	*CXCL5*		*P*-value	*GNRH1*		*P*-value
	expression			expression			expression			expression		
	high	low		high	low		high	low		high	low	
ISUP grade												
1	2	4	>0.1	1	5	>0.1	2	4	>0.1	2	4	>0.1
2–4	3	3		2	4		2	4		3	3	
pT												
T1	5	4	<0.1	3	6	<0.1	3	6	>0.1	5	4	<0.1
T2-T3	0	3		0	3		1	2		0	3	
PN												
N0	4	6	>0.1	2	8	>0.1	4	6	>0.1	5	5	>0.1
N1	1	1		1	1		0	2		0	2	
Pm												
M0	5	6	>0.1	3	8	>0.1	4	7	>0.1	5	6	>0.1
M1	0	1		0	1		0	1		0	1	

ISUP: International Society of Urological Pathology

## Discussion

A total of 128 IRGs differentially expressed in ccRCC tissues compared to normal tissues were identified from TCGA and GEO databases. Subsequently, functional enrichment analysis found that these genes were mainly involved in inflammation and immunity. Univariate Cox, LASSO, and multivariate Cox regression analyses revealed four prognostic hub genes (*RNASE2*, *BIRC5*, *CXCL5*, and *GNRH1*), and a prognosis model was established. We also verified the reliability of the model and found that the four hub genes were closely related to the prognosis of ccRCC patients. To further analyze the reliability of the model, we evaluated the expression of immune infiltrate and tumor mutation load, IRGs, and immunotherapy effect in the two risk groups and found that the four hub genes could predict the immune status of ccRCC patients. These results demonstrated that *BIRC5* expression was significantly higher in ccRCC and tumor cell lines than in paracancer tissues and normal cell lines. The expression of *CXCL5* was significantly higher in tumor tissue than paracancer tissues; however, the expression of *CXCL5* in 786O and HK-2 cells did not differ significantly, which might be because the cancer cell lines do not represent the gene expression of tumor tissues. Also, *GNRH1* and *RENSE2* expressions did not differ significantly between the tissues and cells. Next, we evaluated whether the expression values of these hub genes could affect the prognosis. Due to the short follow-up period, we analyzed the correlation between clinicopathology and the gene expression values and found that tumor grade was correlated with the expression values of the three hub genes. However, due to the small sample size and short follow-up time, the association was not statistically significant (*P*>0.05), necessitating further exploration.

*BIRC5* (Survivin) is strongly expressed in most human tumors and is closely related to tumor progression, recurrence, and chemotherapy resistance [11]. It is also associated with poor prognosis and progression of ccRCC [12, 13]. Current studies have shown that *RNASE2*, a member of the pancreatic ribonuclease family, is a useful prognostic predictor of ccRCC [14]. Among cancer patients, *CXCL5* [15] is closely related to survival time, recurrence, and metastasis and is associated with a poor prognosis in ccRCC [16]. Microslaw et al. [17] demonstrated that the expression levels of chorionic gonadotropin beta subunit and *GnRH1* in the blood of cancer patients might be valuable in indicating tumor metastasis and spread. Wu et al. [18] demonstrated that *GnRH1* and *LTB4R* are prognostic biomarkers for patients with ccRCC.

Reportedly, a risk model can predict the prognosis of patients. For example, Hua et al. [19] combined multiple databases to build a survival model of immune-related prognostic genes, which could accurately predict the prognosis of ccRCC patients. Wan et al. [14] used 7 IRGs to establish a regression model for predicting the prognosis of ccRCC patients and found that the risk model could accurately distinguish patients with different survival outcomes and identify those with high and low mutation loads. Notably, our model could also predict patient prognosis. The four hub genes were considered independent prognostic factors related to the clinicopathological characteristics.

In addition, we analyzed the correlation between the risk model and immune filtration, immune target, and immunotherapy and found that the four genes predicted the immune status. These analyses demonstrated a poor response of the high-risk group to immunosuppression.

In a study by Zhang et al. [20], low levels of resting dendritic cell infiltration were observed in the high mutation load (TMB) group, and high levels of TMB were associated with low survival rates, while low resting dendritic cell infiltration was associated with poor prognosis. Castle et al. [21] reported that dendritic cells activate T cells in patients and increase cytotoxic T cell production, exerting anti-tumor effects. Basile et al. [22] demonstrated that high tumor-infiltrating neutrophils indicated poor overall survival in ccRCC patients. A previous study found that in patients with ccRCC mast cells [23] and high infiltration, the immune treatment may be effective with an improved prognosis. Plasma cells and T cells are crucial in tumor immunity. In the current study, neutrophils, CD4 memory T cells, follicular helper T cells, and regulatory T cells were abundant in the high-risk group, while resting mast cells were low. We further analyzed the prognosis of patients with varying content of immune cells and found that the patients with a high number of resting mast cells had better outcomes than those with low numbers. On the other hand, the patients with low plasma cells, follicular helper T cells, and regulatory T cells had a poor prognosis.

Previous studies showed that the tumor mutation load is positively related to the presence of a novel antigen that significantly enhances the efficacy of immunotherapy. Subsequently, we tested our model and found that the high-risk group had a high mutation load; these results were in agreement with those of Hua et al. [19]. According to the immunotherapy results, we found that the high-risk group had better immunotherapy efficacy. These observations further confirmed the functional correctness of our prognosis model based on the four hub genes.

However, the current study differs from the previous studies in some aspects. First, we combined multiple databases to construct the survival models of four hub genes related to prognosis. Then, we performed internal and external validation, and the qRT-PCR results showed that the two hub genes were differentially expressed in the tumor and normal tissues. Subsequently, we analyzed the immune cell content in different risk groups and the prognosis of patients with varied immune cell content. Finally, we analyzed the differential expression of immune targets, *PD-1* and *CTLA-4*, in the two risk groups in the survival model constructed using the four hub genes and verified the data related to the treatment using the two immune targets downloaded from the public database, proving the accuracy of the model function.

Nevertheless, this study has some limitations. The first is the limited sample size; hence, the findings require further substantiation using multiple samples and multicenter clinical trials. Second, the exact mechanism by which these four genes influence the prognosis of ccRCC patients is yet unknown. In future studies, we need to prospectively follow up on ccRCC patients and detect the correlation of these four genes to confirm the accuracy of the predictive model on the occurrence, development, and prognosis of ccRCC.

## Conclusions

We used four hub genes to construct a prognostic risk model to verify its accuracy. As a prognostic marker, the risk score of this model might distinguish the immune status, immunotherapy effect, and prognosis of patients with different risk scores.

## Supporting information

S1 Table Primer sequences. (TIF)

S1 Fig High- and low-risk grouping and clinical traits. (A–D) Boxplot representing risk scores for different clinical traits.
(TIF)

S2 Fig High- and low-risk assessment grouping and genetics changes. (A-B) Waterfall plot of tumor mutation load in the two risk subgroups. (C) Differential analysis of tumor mutation load in the two risk subgroups.
(TIF)
