# Supplementary figures and images for: Bioinformatics analysis of immune-related prognostic genes and immunotherapy in renal clear cell carcinoma
Source: PLoS One. 2022 Nov 23;17(11):e0272542. doi: 10.1371/journal.pone.0272542 (PMC9683592; doi:10.1371/journal.pone.0272542)

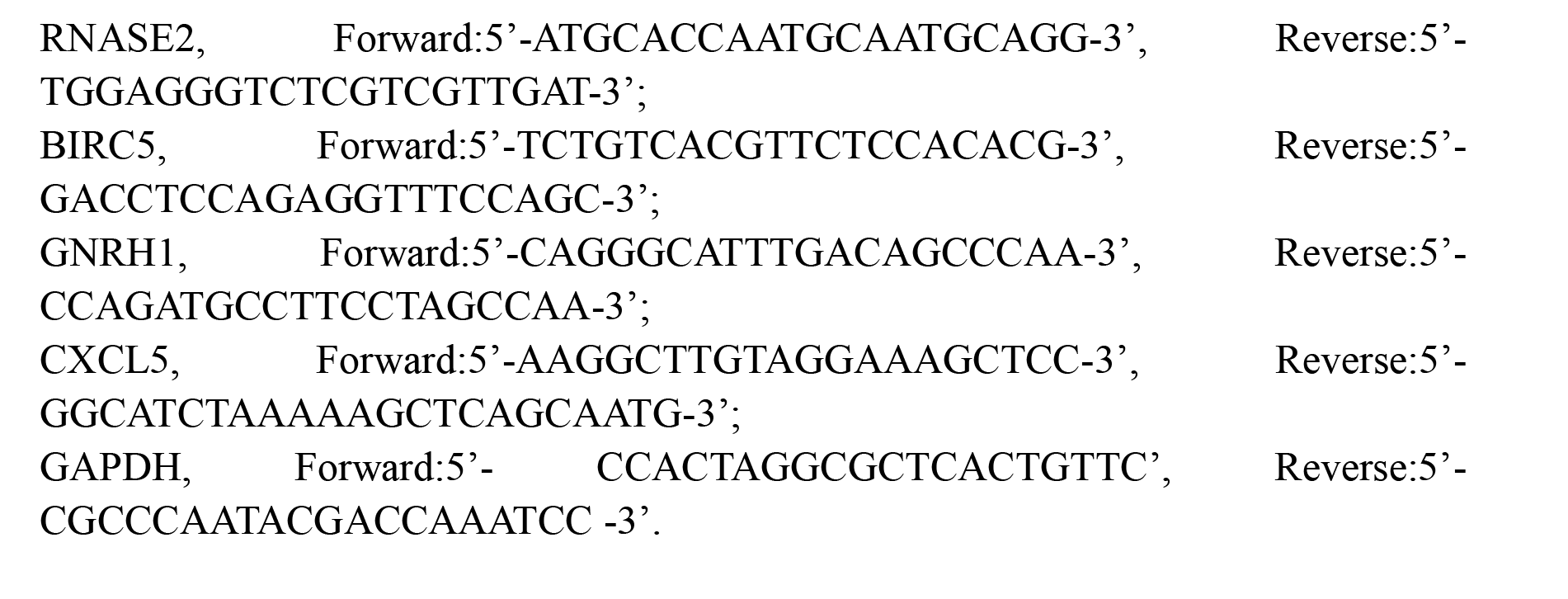

Supplement: S1 Table — (TIF) [file pone.0272542.s001.tif]

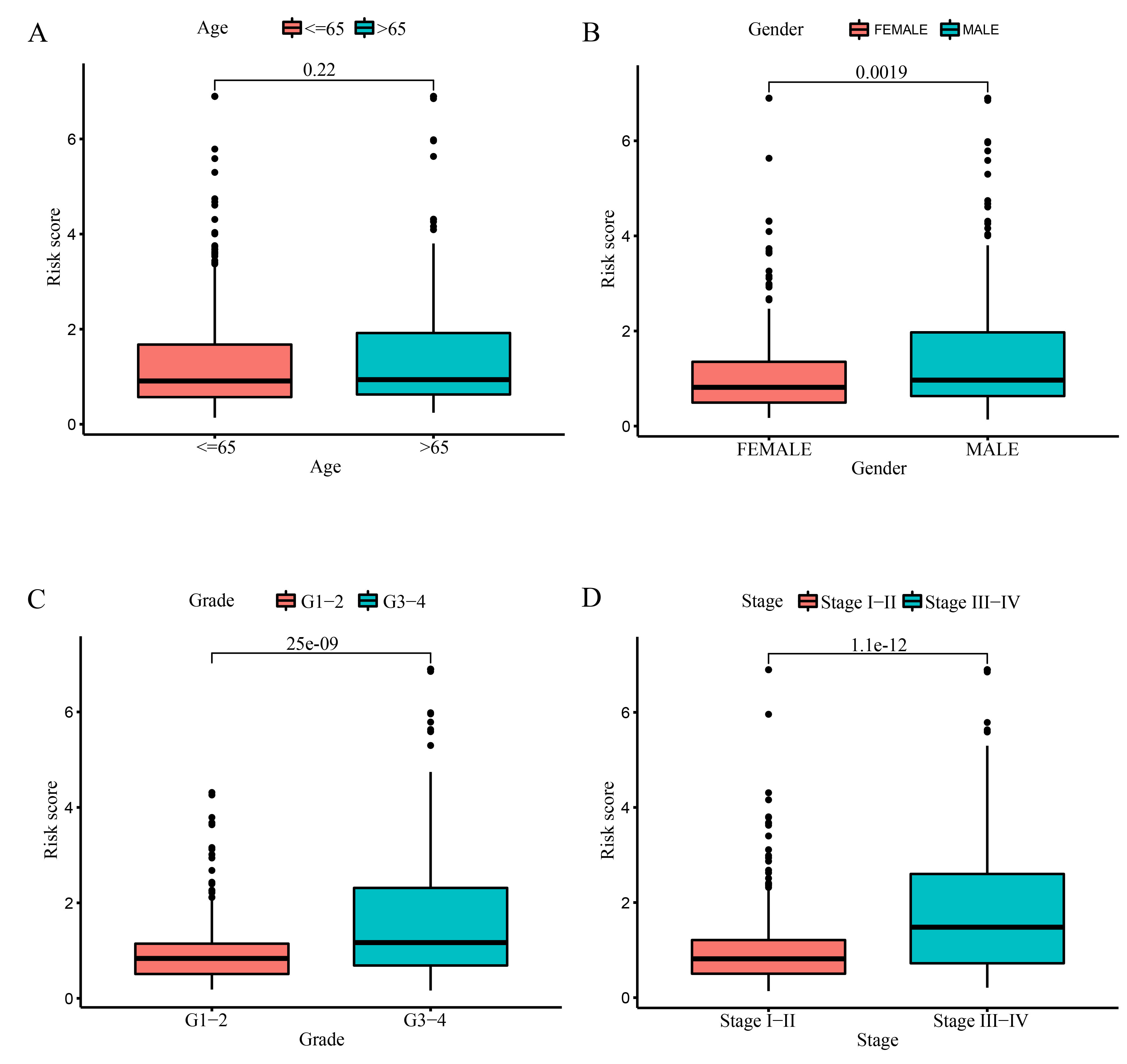

Supplement: S1 Fig — (A–D) Boxplot representing risk scores for different clinical traits. (TIF) [file pone.0272542.s002.tif]

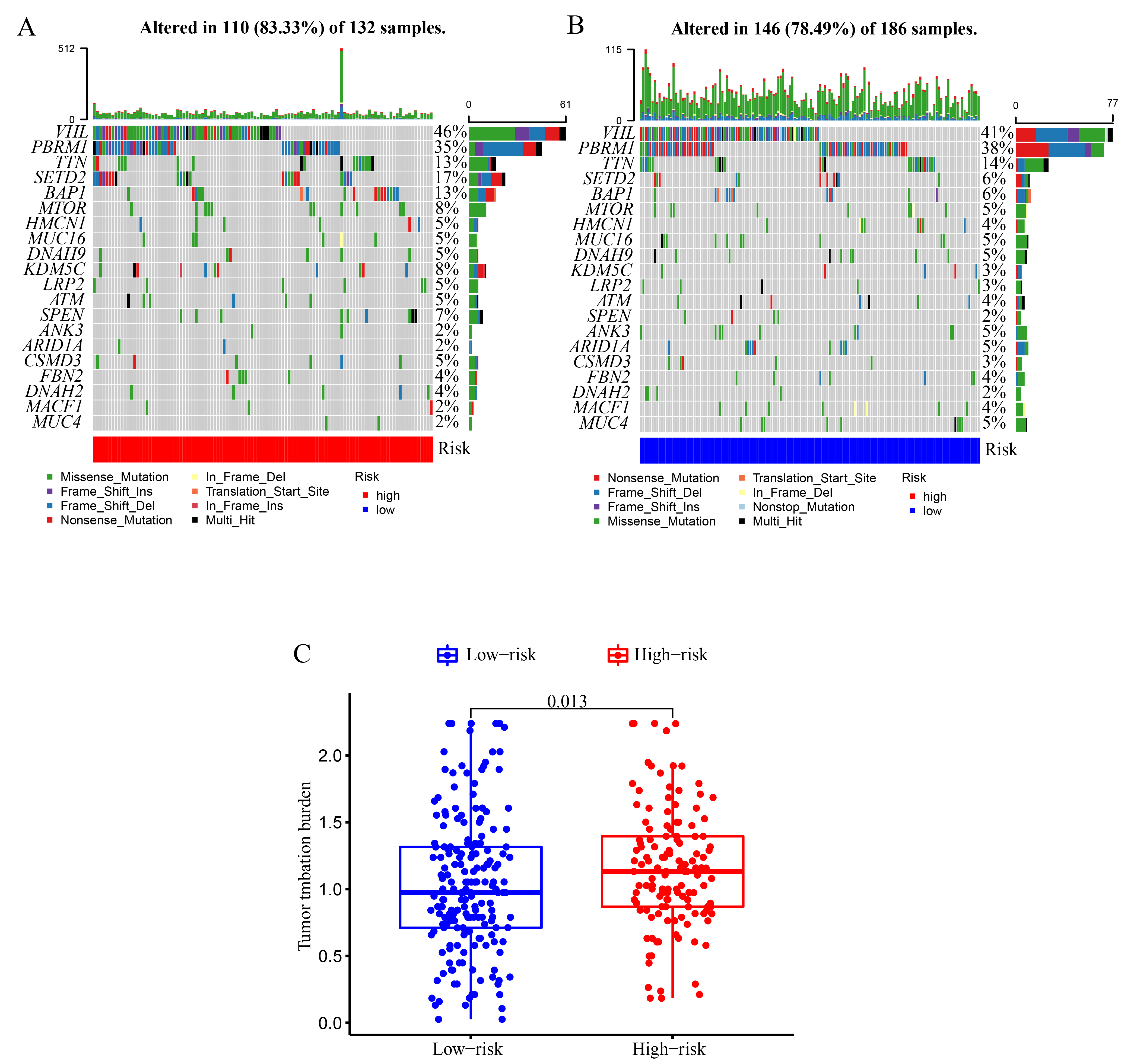

Supplement: S2 Fig — (A-B) Waterfall plot of tumor mutation load in the two risk subgroups. (C) Differential analysis of tumor mutation load in the two risk subgroups. (TIF) [file pone.0272542.s003.tif]
